# Nutritional Status and Information Provided to Polish Cancer Patients Assessed Using the EORTC QLQ-INFO25 Questionnaire

**DOI:** 10.3390/jcm14030697

**Published:** 2025-01-22

**Authors:** Elwira Gliwska, Dominika Głąbska, Zuzanna Zaczek, Jacek Sobocki, Dominika Guzek

**Affiliations:** 1 Department of Food Market and Consumer Research, Institute of Human Nutrition Sciences, Warsaw University of Life Sciences (WULS-SGGW), 159C Nowoursynowska Street, 02-776 Warsaw, Poland; dominika_guzek@sggw.edu.pl; 2 Cancer Epidemiology and Primary Prevention Department, Maria Sklodowska-Curie National Research Institute of Oncology, 15B Wawelska Street, 02-034 Warsaw, Poland; 3 Department of Dietetics, Institute of Human Nutrition Sciences, Warsaw University of Life Sciences (WULS-SGGW), 159C Nowoursynowska Street, 02-776 Warsaw, Poland; dominika_glabska@sggw.edu.pl; 4 Department of Human Nutrition, Faculty of Health Sciences, Medical University of Warsaw, 27 Erazma Ciolka Street, 01-445 Warsaw, Poland; zuzanna.zaczek@wum.edu.pl; 5 Department of General Surgery and Clinical Nutrition, Centre of Postgraduate Medical Education in Warsaw, 231 Czerniakowska Street, 00-416 Warsaw, Poland; jsobocki@mp.pl

**Keywords:** cancer, enteral nutrition, EORTC QLQ-C30, EORTC QLQ-INFO25, malnutrition, quality of life

## Abstract

**Background/Objectives**: Malnutrition in cancer patients may significantly affect various aspects of the quality of life, outcomes, and prognosis, while satisfaction with the information provided may also influence these aspects. This study aims to assess the nutritional status of Polish cancer patients and its association with the level of information received, their potential need for more information, and the resultant quality of life. **Methods**: A cross-sectional study was conducted in 104 cancer patients. Validated European Organization for Research and Treatment of Cancer questionnaires EORTC QLQ-C30 and EORTC QLQ-INFO25 were used, and nutritional assessment was conducted using Subjective Global Assessment (SGA). **Results**: Male patients reported receiving more information than females about the disease, treatment, and care options, as well as greater satisfaction, and a higher overall score. Patients receiving enteral nutrition were more satisfied compared to those not receiving it, even if the scores for the information obtained within the specific areas did not differ, but they still wished to receive more information. Older patients reported higher scores than younger patients, indicating a higher level of information received regarding medical tests and higher satisfaction. The EORTC QLQ-INFO25 global score showed strong or moderate positive correlations with the majority of modules, and the level of information provided significantly influenced satisfaction. **Conclusions**: Female patients, those not receiving enteral nutrition, and young patients were less satisfied with the information received, which may negatively influence their quality of life. Effective communication with patients highlights the need for personalized informational support to enhance quality of life.

## 1. Introduction

The increasing prevalence of cancer is a global problem, as in 2022, the Global Cancer Observatory (by the World Health Organization (WHO)) reported approximately 20 million new cancer cases and 9.7 million cancer-related deaths [1]. At the same time, cancer-related malnutrition is diagnosed in up to 85% of patients with cancer, depending on the affected organ [2], and causes the deterioration of nutritional status in a significant number of cancer patients [3]. The high prevalence of malnutrition in cancer patients results from increased energy requirements, decreased desire to eat and difficulties in eating, disturbed gastrointestinal motility, digestion, and absorption, as well as decreased capacity to utilize nutrients [3]. Furthermore, cancer treatment often causes adverse effects, exacerbating the symptoms of the disease and resulting in an increased risk of malnutrition [4]. Cancers in the gastrointestinal tract, head, or neck can directly impair eating, reducing food intake and the nutritional value of daily diet [5].

The European Society for Clinical Nutrition and Metabolism (ESPEN) clinical guidelines recommend that cancer patients being malnourished or at risk of malnutrition apply nutritional intervention to increase oral intake or to apply enteral nutrition or parenteral nutrition if enteral nutrition is not sufficient or feasible [6]. It is associated with the fact that malnutrition significantly impacts various aspects of well-being, as well as treatment outcomes and prognosis, making appropriate nutritional care essential [7]. This area is associated with the quality of life of cancer patients, being currently indicated among the essential factors to be taken into account while planning adequate therapy, as indicated within the recent systematic review by Shrestha et al. [8], that depending on the general condition of the cancer patient, their quality of life may be more important than the length of life.

Consequently, it is indicated as essential to assess not only clinical symptoms but also subjective patient-reported outcomes associated with quality of life, defined as subjective well-being and including multidimensional (emotional, social, and physical well-being), subjective (patient’s judgments) and non-static aspects (subject to transitions over a patient’s lifetime) [9]. It is associated with the fact that according to a recent systematic review and meta-analysis by Efficace et al. [10], patient-reported outcomes provide independent prognostic information for overall survival across cancer populations and disease stages. Among widely used instruments to assess the quality of life, the most commonly applied are the European Organization for Research and Treatment of Cancer (EORTC) Quality of Life Questionnaire (EORTC QLQ) tools, such as Core 30 (EORTC QLQ-C30), Palliative Care Module (EORTC QLQ-C15-PAL), Head and Neck Cancer Module (EORTC QLQ-H&N35), Gastric Cancer Module (EORTC QLQ-STO22), or many other [10].

Among the factors associated with the quality of life of patients with chronic diseases are those resulting from their expectations and previous experiences [11]. However, it may be moderated by the communication and provided information, which may reduce feelings of unpredictability and increase understanding of the applied treatment, as well as increase the trust by the feeling of being in a form of relationship with health professionals [12]. Taking this into account, the EORTC also developed the EORTC QLQ-INFO25, a tool that may be used to assess the perception of information received by cancer patients based on a simple question about how much information they received about various aspects of the disease, medical tests, treatment, and other [13]. This tool identifies areas where patients may face challenges due to inadequate information, which provides valuable insights to improve communication between healthcare providers and patients [14]. Since this questionnaire also includes a question about overall satisfaction with the information provided (though not in specific areas), it helps identify whether the patient needs more information. This is important, as some studies suggest that even patients who are not properly informed may still be satisfied with the information they received and do not wish to receive more [15].

Considering this, the presented study aims to assess the nutritional status of Polish cancer patients and its association with the level of information they receive, their potential need for additional information, and the resultant quality of life. Additionally, the study explored the association of sex, age, applied enteral nutrition, and global characteristics with the information patients receive to provide a comprehensive understanding of the findings. It was supposed that the listed factors may potentially cause medical healthcare providers to provide more information or provide it differently than in the case of other patients.

## 2. Materials and Methods

### 2.1. Basic Information About the Study

**Figure 1:**
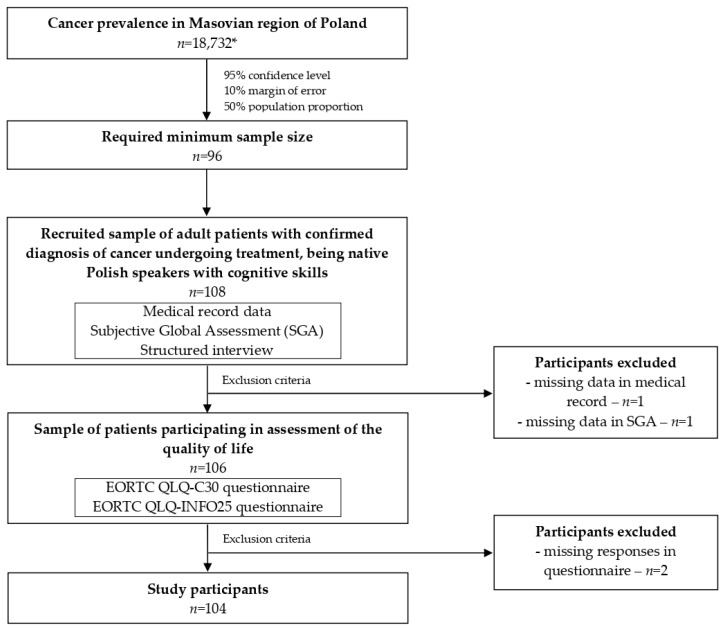
Study design and data collection. * based on the National Cancer Registry of Poland for 2021 [16]; EORTC QLQ-C30—European Organization for Research and Treatment of Cancer Quality of Life Questionnaire-Core 30; EORTC QLQ-INFO25 C30—European Organization for Research and Treatment of Cancer Quality of Life Questionnaire-Information 25.

The presented cross-sectional study was conducted on a population of cancer-diagnosed patients recruited in (1) the Gastroenterology Unit of the Oncological Hospital in Warsaw and (2) the Polish Outpatient Clinic for Parenteral and Enteral Nutrition in Warsaw. The study design and data collection phases are presented in Figure 1. The participants included in this study received all necessary information about the aim of the study and its procedures in order to be able to provide informed consent to participate in the study.

The presented study was planned based on the Declaration of Helsinki and its guiding principles. The Bioethics Committee of the Center for Postgraduate Medical Education in Warsaw provided consent for the study (14 July 2021, 116/2018), and the study participants were recruited in the period of January 2022–September 2023.

### 2.2. Studied Population and Study Procedures

The studied population included 104 cancer patients, recruited based on purposive sampling, while the following inclusion criteria were applied:

- Adult patients (age > 18 years);
- Diagnosed active cancer;
- Undergoing cancer treatment;
- Native speakers of the Polish language with adequate linguistic abilities to communicate and recognize even minor differences in meaning;
- Cognitive skills not suggesting any cognitive decline;
- Agreement for participation in the study.

The exclusion criteria were applied as follows:

- Pregnancy;
- Lactation;
- Any missing data in the medical record;
- Any missing answers in the questionnaire;
- Nonsensical responses in the questionnaire;
- Patients with psychiatric disorders;
- Patients taking neuropsychiatric medications;
- No written informed consent for participation in the study.

The study procedure was based on gathering data while using validated questionnaires as follows:

- EORTC QLQ-C30 questionnaire;
- EORTC QLQ-INFO25 questionnaire.

The data were gathered within the structured interview conducted by a researcher, while one experienced dietician gathered data from a whole studied group to reduce potential bias.

The EORTC QLQ-C30 questionnaire was developed by the EORTC and it is currently available in over 120 language versions [17]. It has been validated and shown to be characterized by good content validity for assessing functional health, symptom burden, and health-related quality of life in patients with various cancers [18].

Among the various language versions, Polish version was also validated and described as a reliable and valid tool to assess health-related quality of life in patients with esophagi-gastric cancer [19]. Tomaszewski et al. [19] noted that the EORTC QLQ-C30 scales demonstrate Cronbach’s alpha values of 0.7 or higher, indicating good internal consistency. All multi-item scales present strong own-scale correlations, with the lowest correlation observed for the physical functioning scale. For test–retest reliability, the Intraclass Correlation Coefficients (ICCs) were used, which ranged from 0.82 to 0.91, demonstrating good reliability and reproducibility [19]. The Polish version of the EORTC QLQ-C30 questionnaire is commonly used in patients with various cancers [20,21].

The EORTC QLQ-C30 questionnaire is a 30-item tool developed for patients with various cancers, which allows for the comparison of populations with various cancers, and it may be applied as an additional tool to improve the sensitivity and specificity of the quality of life assessments [22]. It is currently one of the most often used tools to assess patient-reported outcomes in oncology [23]. It was developed in the 90s as a self-administered tool to be completed independently by patients, containing understandable, easy questions [24].

In the most recent EORTC QLQ-C30 3.0 version, among 30 items, there are 24 organized into 9 scales, as follows: (1) physical functioning (5 items), (2) role functioning (2 items), (3) cognitive functioning (2 items), (4) emotional functioning (4 items), (5) social functioning (2 items), (6) global health status/quality of life (2 items), (7) fatigue (3 items), (8) pain (2 items), and (9) nausea and vomiting (2 items). Scales (1)–(5) are defined as 5 functional scales, scale (6) is defined as an independent scale allowing for general assessment, and scales (7)–(9) are defined as symptomatic scales. Additionally, within the EORTC QLQ-C30 questionnaire, there are 6 single-item questions about the following areas: (1) dyspnea, (2) insomnia, (3) loss of appetite, (4) constipation, (5) diarrhea, and (6) financial difficulties [25]. The questions are formulated about various symptoms, feelings, and functioning, while they are either for general assessment or for the assessment during the previous week. The 4-category scale is used with defined frequencies, as follows: not at all, a little, quite a bit, and very much, and for only 2 items of global health status/quality of life, the 7-category scale is used, from very poor to excellent. Based on the results, the linear transformation of the raw score is applied to standardize and obtain a score ranging from 0 to 100 for all the scales and single-item questions, while the higher score represents better functioning [26].

The EORTC QLQ-INFO25 questionnaire was developed by EORTC, and it is currently available in over 30 language versions [27]. This questionnaire has been validated and described as demonstrating a good level of reliability and validity for the assessment of information received by patients with various cancers [13]. Among the various language versions, the Polish version was also validated and found to exibit a good level of internal consistency and reliability, making it suitable for use in clinical settings and scientific research [28].

The EORTC QLQ-INFO25 questionnaire is a 25-item tool developed for patients with various cancers to assess the information they have received about their disease and its treatment [29]. It also allows for a comparison between populations of patients with different types of cancers [30]. In the EORTC QLQ-INFO25, among 25 items, there are 19 organized into 6 scales, as follows: (1) disease (4 items), (2) medical tests (3 items), (3) treatment (6 items), (4) other services (4 items), (5) different places of care (1 item), and (6) things patients can do to help themselves (1 item) [31]. The questions are formulated about various topics to specify how much information the patient received, using the 4-category scale as follows: not at all, a little, quite a bit, and very much. Additionally, the EORTC QLQ-INFO25 includes: (1) two yes–no questions about receiving written information and information on CD/video, (2) one question about satisfaction with the amount of information received rated on the 4-category scale (not at all, a little, quite a bit, and very much), (3) two yes–no questions about willingness to receive more or less information along with options of topics on which more or less information is desired, and (4) one question about if overall the information received was helpful using the 4-category scale: not at all, a little, quite a bit, and very much [29]. Based on the results, the linear transformation of the raw score is applied to standardize and obtain a score ranging from 0 to 100 for all the scales and single-item questions, while the higher score represents more information [31].

Additional information about each patient, their age, disease, course of the disease, and applied treatment, as well as nutritional status, were obtained from the medical record cards. The information about nutritional status included:

- Body Mass Index (BMI) (calculated based on medical record card data about body mass and height) [32];
- Body mass change (calculated based on medical record card data about current body mass and body mass 6 months before);
- Subjective Global Assessment (SGA), being a valid tool for the nutritional assessment of hospitalized clinical patients [33], obtained from the medical records.

Patients also completed a simple questionnaire to describe their basic sociodemographic characteristics. They were asked about their educational background, employment, place of residence, and subjective economic status assessment.

Based on the gathered data, patients were stratified based on:

- BMI—stratified into categories of underweight, normal body weight, and overweight according to commonly applied categories by WHO [32];
- SGA—stratified into categories of A (well-nourished), B (moderately malnourished or suspected of being malnourished), and C (severely malnourished) according to commonly applied categories by Detsky et al. [34];
- Global Health Status—stratified into categories of good (score > 50) and bad quality of life (score ≤ 50), based on the cut-off of 50 by Diouf et al. [35];
- Age—stratified into categories of early-onset cancer (EOC) (19–49 years), as defined by Ugai et al. [36], middle age (50–69 years) and older (70–82 years), as commonly indicated in cancer studies [37].

### 2.3. Statistical Analysis

Evaluation of the quality of data obtained while using the applied questionnaires included the linear transformation of the raw score, internal consistency assessment conducted based on the ceiling and floor effects [38], and Cronbach’s alpha coefficient [39]. As commonly assumed, a value higher than 15% for either the ceiling or floor effect was interpreted as an unsatisfactory level of floor or ceiling effect, respectively [40], while a Cronbach’s alpha coefficient lower than 0.7 was interpreted as not satisfactory, as recommended by DeVellis [41].

The normality of the distribution was evaluated using the Shapiro–Wilk test. Comparisons between groups were conducted using the χ^2^ test (for categorical variables), Student’s *t*-test (for comparison of 2 subgroups for parametric distributions), the Mann–Whitney U test (for comparison of 2 subgroups for non-parametric distributions), analysis of variance (ANOVA) test (for comparison of more than 2 subgroups for parametric distributions), or Kruskall–Wallis ANOVA test (for comparison of more than 2 subgroups for non-parametric distributions). The analysis of correlation was conducted using the Pearson correlation (for parametric distributions) and the Spearman rank correlation (for non-parametric distributions), while the correlations were interpreted as strong for |R| > 0.7, moderate for |R| ∈ (0.5; 0.7>, fair for |R| ∈ (0.3; 0.5>, or poor for |R| ≤ 0.3 [42].

Effect size was calculated using the Cohen’s d, eta-squared (η^2^), and Cramer’s V. Cohen’s d was used to describe the standardized mean difference of an effect and to classify effect sizes as small (d = 0.2), medium (d = 0.5), and large (d ≥ 0.8). Eta-squared was calculated as the sum of squares between groups divided by the total sum of squares of the dependent variable. Eta-squared values indicated a small (η^2^ = 0.01), medium (η^2^ = 0.06), and large (η^2^ = 0.14) effects [43]. Cramer’s V was applied for categorical data, ranging from 0 (no association) to 1 (perfect association) [44].

The level of *p* ≤ 0.05 was considered statistically significant. The statistical analysis was performed using Statistica 13.3 (StatSoft Inc., Tulsa, OK, USA), and the JASP version 0.14.0.0 statistical software (Department of Psychological Methods University of Amsterdam, Amsterdam, The Netherlands, https://jasp-stats.org/).

## 3. Results

Table 1 presents the basic characteristics of the studied group of cancer patients, including age, BMI, and declared weight loss in the last six months. The median age was 60 years, and the median BMI was 23.95 kg/m^2^. Within the studied group, there were patients characterized by underweight (*n* = 11; 10.6%), normal body weight (*n* = 50; 48.1%), and overweight (*n* = 43; 41.3%), while the majority of them declared body weight loss (*n* = 70; 67.3%). Within the subgroup declaring weight loss, the median weight loss was 10% of body weight.

Table 2 presents the sociodemographic characteristics of the studied group of cancer patients. Most of the patients were women, representing over half of the group. Nearly half of the patients had higher education, followed by those with general secondary education and a smaller share declaring vocational secondary and primary education. Regarding employment status, most patients were retired, with one in four holding a full-time permanent position. A smaller number were employed part-time, held temporary positions, received a pension, or were not currently working. The patients came from various residential backgrounds, with the largest group residing in rural areas or cities with more than 500,000 inhabitants. Regarding economic status, most patients reported their situation as either good or rather good, while others reported it as bad or rather bad or could not assess it.

Table 3 presents the distribution of cancer sites identified in the studied group of cancer patients. The data highlight a predominance of cancers affecting the head and neck region, with laryngeal cancer (C32) being the most frequently diagnosed and representing 18.3% of cases, while the other cancer sites in the head and neck region were floor of mouth (C04), lip, oral cavity, and pharynx (C14), and esophagus (C15), collectively constituting 51.9% of cases in the studied group for head and neck cancers. Among gastrointestinal cancers, the diagnoses were made for stomach (C16), small intestine (C17), colon (C18), liver and intrahepatic bile ducts (C22), other parts of the biliary tract (C24), and pancreas (C25), collectively representing 17.3% of cases in the studied group, with stomach cancer being the most frequent among these (4.8%). Among the other cancers, there were those of bronchus and lung (C34), breast (C50), corpus uteri (C54), and prostate (C61), collectively representing 30.8% of cases in the studied group, with breast (C50) and ovary cancer (C56) as the most predominant, accounting for 8.7% of cases each.

Table 4 presents the nutritional characteristics of the studied group of cancer patients. Even if the BMI assessment did not reveal any significant body mass problems, a share of two-thirds of patients experienced weight loss, which corresponded to over 70% of patients classified as being already malnourished or at risk of malnutrition. Additionally, most patients reported a reduced food intake, and over 70% assessed their quality of life as low. However, enteral nutrition was not introduced in more than 76% of the studied patients.

Table 5 presents the sociodemographic and health-related characteristics of the studied group of cancer patients stratified by the Subjective Global Assessment category. No statistically significant differences were found between the compared subgroups for most sociodemographic features, and only place of residence and economic status differed. The majority of individuals classified as severely malnourished were characterized by bad/rather bad economic situations and were living in rural areas, while those classified as well-nourished or moderately malnourished were characterized by good/rather good economic situations and were living in cities of over 100,000 inhabitants. At the same time, there were statistically significant differences between all health-related characteristics. The majority of individuals classified as severely malnourished or moderately malnourished declared weight loss, reduction in food intake, and low quality of life, while the majority of those classified as well-nourished declared no weight loss, no reduction in food intake, and high quality of life. Similarly, a higher share of underweight individuals and those subjected to enteral nutrition was observed for individuals classified as severely malnourished or moderately malnourished than for those classified as well-nourished.

Table 6 presents the summary of the descriptive statistics and reliability metrics for scales and individual items of the EORTC QLQ-INFO25 questionnaire. For all assessed scales and individual items, Cronbach’s alpha was satisfactory, suggesting good internal consistency. However, the ceiling and floor effect revealed unsatisfactory results for information about other services, different places of care, and things patients can do to help themselves, and the floor effect revealed unsatisfactory results for information about medical tests.

Table 7 presents the number and percentage of patients reporting low satisfaction on the EORTC QLQ-INFO25 scales. Patients reported dissatisfaction in various areas, with the highest dissatisfaction observed in information about other services (74%) and different places of care (72%), which were recognized as requiring more focus. Slightly less dissatisfaction was noted in information about things patients can do to help themselves (66%), treatments (38%), medical tests (30%), and the disease itself (31%). While these areas show comparatively lower percentages, they still highlight a considerable portion of patients experiencing very low satisfaction (scoring below 50 points in the respective subscales) and merit targeted improvements.

Table 8 presents the scales and individual items of the EORTC QLQ-INFO25 questionnaire of the studied group of cancer patients stratified by sex. Male patients reported higher scores than female patients, indicating a higher level of information obtained regarding the disease, treatments, and different places of care. Similarly, regarding the satisfaction with the information received, male patients expressed higher satisfaction than female patients. This resulted in a higher global score observed for male patients than for female patients.

Table 9 presents the scales and individual items of the EORTC QLQ-INFO25 questionnaire of the studied group of cancer patients stratified by application of enteral nutrition. Regarding satisfaction with the information received, patients receiving enteral nutrition expressed a higher level of satisfaction than those not receiving enteral nutrition, even if the scores for the information obtained within the specific areas did not differ. However, at the same time, a higher score was declared by patients receiving enteral nutrition than those not receiving it, regarding wishing to receive more information.

Table 10 presents the scales and individual items of the EORTC QLQ-INFO25 questionnaire of the studied group of cancer patients stratified by age. Older patients reported higher scores than younger patients, indicating a higher level of information obtained about medical tests. Similarly, regarding satisfaction with the information received, older patients expressed higher satisfaction than younger patients.

Table 11 presents the scales and individual items of the EORTC QLQ-INFO25 questionnaire of the studied group of cancer patients stratified by the SGA categories. The results do not show statistically significant differences between the SGA groups across most scales and items.

Supplementary Table S1 presents the correlations between scales and individual items of the EORTC QLQ-INFO25 questionnaire in the studied group of cancer patients. Global score showed strong or moderate positive correlations with the majority of other modules, including ratings on the scales associated with information about the disease, information about treatments, satisfaction with information received (strong correlations), information about medical tests, information about other services, information about different places of care, and information about things a patient can do to help themselves (moderate correlations). It may be indicated that more information provided within the indicated areas was associated with a higher global score. Moreover, ratings on the scales related to information about the disease and information about treatments presented a strong correlation, suggesting that patients who feel well-informed about their disease are also likely to feel informed about available treatments.

At the same time, moderate positive correlations were observed between satisfaction with the information received and the ratings on the scales associated with the following: information about the disease, information about medical tests, information about treatments, and information about other services. This suggests that satisfaction was significantly influenced by the level of information provided in these areas. Moreover, a moderate positive correlation was observed between satisfaction with information received and the rating on the scale associated with the statement that, overall, the information has been helpful.

Other moderate positive correlations were stated between the ratings on the scales associated with information in the following areas: information about the disease with information about medical tests and information about other services; information about medical tests with information about treatments; information about other services with information about different places of care and information about things patient can do to help themselves; information about different places of care with information about things patient can do to help themselves.

Supplementary Table S2 presents the correlations between scales and individual items of the EORTC QLQ-INFO25 and the EORTC QLQ-C30 questionnaire in the studied group of cancer patients. The analysis revealed poor or no correlations. The poor correlations were observed between the ratings on the scale associated with information about medical tests with scores for role and social functioning scales (negative correlations), as well as dyspnea and appetite loss single-item measures (positive correlations); the ratings on the scale associated with information about treatments with scores for vomiting/nausea and dyspnea single-item measures (positive correlations); the ratings on the scale associated with information about different places of care with score for emotional functioning scale (positive correlation); the ratings on the scale associated with wishing to receive more information with score for emotional functioning scale (positive correlation); the ratings on the scale associated with the statement that overall the information has been helpful with scores for role functioning scale (negative correlation), as well as pain and vomiting/nausea single-item measures (positive correlations); the average of all the scales/single-item measures with score for vomiting/nausea single-item measures (positive correlation).

## 4. Discussion

The presented study examined the nutritional status of Polish cancer patients, as well as their quality of life and perception of and satisfaction with the information they received. The obtained data highlight that cancer patients face not only a high risk of symptom burden and reduced quality of life but also significant unmet informational needs throughout their treatment. However, it should be emphasized that informational needs vary among patients and are influenced by several factors, so they should be addressed individually.

### 4.1. Patient Characteristics and Nutritional Status

The majority of patients were middle-aged and had a moderate BMI. Most of them reported weight loss in the past six months and reduced food intake, indicating common nutritional challenges within this group, even though their BMI suggested normal body mass or even overweight. Over 70% of patients in this study were malnourished or at risk of malnutrition, according to the SGA categories.

In general, the percentage of cancer patients with diagnosed malnutrition depends on the cancer site, stage of the disease, and treatment [45]. Simultaneously, a study by Muscaritoli et al. [46], conducted in Italy, indicated that nearly half of cancer patients are at risk of malnutrition at their first oncology visit, with the severity of malnutrition positively correlated with the stage of cancer. The studies also revealed that weight loss significantly impacts various aspects of well-being, and even 6% of body weight loss predicts reductions in treatment response, survival, and quality of life [47]. In the study by Rios et al. [48], poorer nutritional status was significantly linked to a decline in certain functional domains of QoL in elderly cancer patients. However, it should be noted that age also influences various domains of QoL, and thus, the impact of body mass loss may differ in younger cancer patients. Moreover, the patient’s malnutrition is related to psychological factors, e.g., symptoms of anxiety and depression [49].

In the presented study, among the assessed factors associated with nutritional status were BMI, weight loss, reduction of food intake, applied enteral nutrition, and SGA category. However, it must be emphasized that the indicated factors do not cover the whole area associated with malnutrition in cancer patients. The cancer cachexia, being defined as a multifactorial disease characterized by weight loss via skeletal muscle and adipose tissue loss, an imbalance in metabolic regulation, and reduced food intake, may be recognized based on various criteria [50]. The international consensus criteria by Fearon et al. [51], supported by the European Palliative Care Research Collaborative (EPCRC), indicated weight loss in the absence of simple starvation or decreased BMI accompanied by weight loss, or appendicular skeletal muscle index loss accompanied by weight loss. The proposal by the SCRINIO Working Group, based on the database of a multicenter prospective investigation screening of the nutrition risk of oncology patients [52], indicated weight loss accompanied by at least one symptom of anorexia, fatigue, or early satiation. The criteria by Evans et al. [53], developed within the cachexia consensus conference, indicated weight loss accompanied by at least three of the following criteria: decreased strength, fatigue, anorexia, low fat-free mass, or abnormal biochemistry (increased inflammatory markers, anemia, or low serum albumin).

Taking this into account, it must be indicated that even if body weight (or BMI) and weight loss are the major factors associated with cachexia, there are also factors associated with sarcopenia, namely muscle strength, muscle quantity and quality, and physical performance, which cover the operational definition of sarcopenia, developed by European Working Group on Sarcopenia in Older People (EWGSOP) [54]. It should be noted that BMI does not always reflect skeletal muscle, and patients with the same BMI can have different proportions of lean and fat mass [55]. However, a recent systematic review and meta-analysis of 73 cohort studies by Wen et al. [56] indicated that BMI, weight change, and cancer prognosis are significantly correlated, while factors such as muscle–fat ratio, fat-free BMI, and visceral adiposity have not been systematically discussed in terms of significance in cancer survival, and this issue still needs to be explored.

Even if for cancer survival the role of body mass was proven, while the role of muscle mass was still not explored, it was emphasized by Landi et al. [57] that malnutrition needs to be addressed as a muscle-related disorder. Moreover, nutritional interventions may provide energy and amino acids for protein synthesis, but in certain cachectic conditions, they may maintain body weight but not muscle mass [58]. Therefore, low muscle mass is considered a component of the diagnosis of malnutrition considered by the Global Leadership Initiative on Malnutrition (GLIM) criteria [59]. Muscle mass may be assessed by computed tomography (CT), dual-energy X-ray absorptiometry (DXA), or bioelectrical impedance analysis (BIA) [60]. However, due to certain limitations of these techniques, questionnaire assessments and body weight loss remain important tools in clinical practice for evaluating malnutrition in cancer patients, particularly in studies involving patients from diverse healthcare facilities.

It should be mentioned that ESPEN emphasizes the importance of nutritional interventions to improve oral intake in cancer patients who can eat but are either malnourished or have a risk of malnutrition. These interventions include providing dietary guidance, addressing symptoms and conditions that hinder food consumption, and offering oral nutritional supplements [6].

### 4.2. Nutrition and the EORTC QLQ-INFO25

Nutrition in cancer patients plays a critical role in alleviating cancer cachexia symptoms, such as muscle wasting. Oral nutritional interventions in cancer patients may improve energy and nutrients intake, but they may also improve some other aspects of the quality of life in patients who are malnourished, although they may not necessarily impact survival [61]. In the context of the impact of received information on cancer patients, the study by Bourdel-Marchasson et al. [62] found that early dietary counseling was effective in increasing nutritional intake, but no beneficial effect on mortality rate was stated, highlighting the complexity of the relationship between nutritional interventions and overall survival in cancer patients.

A systematic review by Parsons et al. [63] regarding nutrition applied to improve cancer health outcomes revealed that there are studies indicating improvements in body weight or body composition obtained after providing postoperative nutritional support, as well as studies reporting a reduction in adverse events or length of hospital stay obtained through various enteral and oral nutrition support interventions. At the same time, this systematic review [63] reported that some results were inconsistent, with some studies showing benefits, while others found no difference in adverse events, length of hospital stay, readmissions, emergency room visits, and survival. However, Parsons et al. [63] emphasized that heterogeneity of populations, interventions, comparators, and outcomes precluded aggregation and they stated that coordinated efforts are needed to create detailed frameworks that explain the mechanisms of nutritional interventions most relevant to healthcare providers and patients.

As recommended by ESPEN, higher levels of nausea, appetite loss, and nutritional status disturbances are clear indications to implement enteral feeding; however, it may severely impact overall well-being [6]. While enteral nutrition is crucial for patients unable to eat orally, it is essential to emphasize the need for careful monitoring. If a decision has been made to provide nutrition, enteral nutrition is recommended when oral nutrition remains inadequate despite nutritional interventions such as counseling or oral nutritional supplements. Parenteral nutrition should be considered if enteral nutrition is insufficient or not feasible. However, proper nutritional care can improve various clinical outcomes, but it is often emphasized that it must be part of a holistic approach, such as in the Enhanced Recovery After Surgery (ERAS) protocol [64] or comprehensive care for palliative cancer patients [65].

This study compared the EORTC QLQ-INFO25 module areas based on the type of nutrition patients received. Overall, patients receiving enteral nutrition reported higher satisfaction with the information provided than those without enteral nutrition, but the cause and effect were not defined here. Individuals on enteral nutrition also expressed a stronger desire for additional information. The global scores, reflecting the overall assessment of received information, were significantly higher for the enteral nutrition group, indicating they felt better informed and more satisfied with the information. This may potentially have resulted from additional time dedicated to enteral nutrition training provided to enterally fed patients. Additionally, the complexity of the procedure may require more information.

### 4.3. Demographic Variability in Patient Satisfaction and Information Needs

#### 4.3.1. Sex and the EORTC QLQ-INFO25

Sex-based differences in health literacy and seeking health information have been observed in a number of studies [66,67] confirming sex-based discrepancies. The observed differences may result from different cultural contexts. In the study of Lee et al. [64], women were more likely to use the Internet for health-related information seeking, while in the study of Wang et al. [68], women were less involved in seeking health literacy than men. According to the study by Heizomi et al. [67], while women demonstrated lower levels of health literacy compared to men, their health knowledge was higher. Similarly, in this study, the EORTC QLQ-INFO25 module revealed significant sex differences in perceived information and satisfaction levels. Male participants reported feeling better informed about their disease, treatment options, and available care settings compared to female participants, alongside expressing higher satisfaction with the information provided. Men also demonstrated a greater overall desire for additional information. Overall, men achieved a higher global information satisfaction score, underscoring distinct sex-based perceptions regarding healthcare information and satisfaction. It is observed that individuals satisfied with the information scored significantly higher on global health status (from the EORTC QLQ-C30 questionnaire) [69].

The same results, indicating that women were less satisfied, but unlike in this study, did not desire more information than men, were found in the study by Arraras et al. [13]. Even though some studies suggest satisfaction levels depend on sociodemographic characteristics [13,14,15], the findings related to sex differences may be influenced by the specific study group. Even some studies [14,15] revealed no relationship between satisfaction with the information and sex.

There is still limited research on how men find information and cope with a cancer diagnosis. It should be mentioned that support groups for cancer patients mainly focus on specific types of cancer, especially breast cancer, which may help them cope better in such situations. Nevertheless, healthcare professionals should be aware that men and women may cope with cancer differently, seek information in different ways, and have different informational needs [70].

However, the study leaves the question of why men have a lower need for information than women, and it is necessary to consider other factors, such as psychological ones, as well. Further investigation into these psychological factors could clarify the observed sex differences in information needs and satisfaction levels.

#### 4.3.2. Age and the EORTC QLQ-INFO25

This study compared information and satisfaction scores across different age groups. Older patients reported feeling more informed about medical tests and were generally more satisfied with the information provided than younger groups. For disease-related information and overall satisfaction, older participants scored slightly higher than younger and middle-aged groups, approaching statistical significance. This is in line with the study by Arraras et al. [71] and Bergenmar et al. [69], where the authors stated that younger individuals were less satisfied with the information received; however, in the last study, the participants were women. As we know from the study by Arraras et al. [13], sex is also a factor that can influence the level of satisfaction with the information received.

### 4.4. Strengths and Limitations of the Study

While the study provides valuable insights into the relationship between nutritional status and informational needs among cancer patients, several limitations should be acknowledged. First, the sample size may limit the generalizability of the findings, particularly concerning specific cancer types and subgroups. Future research should aim to include a larger, more diverse sample across various cancer types and stages. Second, the study’s cross-sectional design restricts the ability to draw causal conclusions between nutritional status, information satisfaction, and quality of life. Longitudinal studies are needed to understand better how changes in nutritional status over time influence patients’ informational needs and overall health outcomes. Regarding informational needs, the study identified some differences in satisfaction and perceived information, but it did not explore the underlying psychological factors that may influence these differences, such as coping mechanisms. Future studies could investigate the psychological aspects of information-seeking behavior and satisfaction to provide further insights.

## 5. Conclusions

In the presented study, female patients, those not receiving enteral nutrition, and younger patients were less satisfied with the information received, which may negatively influence their quality of life. The findings emphasize the role of effective communication with patients, highlighting the need for personalized informational support to enhance the quality of life.

## Figures and Tables

**Table 1:** The basic characteristics of the studied group of cancer patients (*n* = 104).

	Mean ± SD	95% CI	Median * (IQR)
Age, years	57.94 ± 14.27	48.5–70	60 (21.5)
BMI, kg/m^2^	24.6 ± 4.81	23.67–25.54	23.95 (3.95)
% of weight loss in individuals declaring weight loss during the last six months (*n* = 69)	11.37 ± 6.92	9.72–13.02	10 (8.0)

* The non-parametric distribution (Shapiro–Wilk test; *p* < 0.05); BMI—Body Mass Index; SD—standard deviation; IQR—interquartile range.

**Table 2:** Sociodemographic characteristics of the studied group of cancer patients.

	Characteristics	Number (%)
Sex	Woman	55 (52.9%)
	Man	49 (47.1%)
Education background	Higher	47 (45.2%)
	General secondary	36 (34.6%)
	Vocational secondary and primary	21 (20.2%)
Employment	Full-time permanent	27 (26.0%)
	Part-time permanent	4 (3.8%)
	Full or part-time temporary	5 (4.8%)
	Pension	7 (6.7%)
	Retirement	49 (47.1%)
	Not employed	12 (11.5%)
Place of residence	City over 500,000 inhabitants	31 (29.8%)
	City 100,000 to 500,000 inhabitants	5 (4.8%)
	City 20,000 to 100,000 inhabitants	21 (20.2%)
	Town up to 20,000 inhabitants	13 (12.5%)
	Rural area	34 (32.7%)
Economic status	Good	25 (24%)
	Rather good	40 (38.5%)
	Rather bad	17 (16.3%)
	Bad	10 (9.6%)
	Difficult to say	12 (11.5%)

**Table 3:** Distribution of cancer sites identified in the studied group of cancer patients.

ICD-10 Code	Cancer Site	Number (%)
C04	Malignant neoplasm of floor of mouth	10 (9.6%)
C14	Malignant neoplasm of other and ill-defined sites in the lip, oral cavity, and pharynx	15 (14.4%)
C15	Malignant neoplasm of esophagus	10 (9.6%)
C16	Malignant neoplasm of stomach	5 (4.8%)
C17	Malignant neoplasm of small intestine	1 (1.0%)
C18	Malignant neoplasm of colon	4 (3.8%)
C22	Malignant neoplasm of liver and intrahepatic bile ducts	4 (3.8%)
C24	Malignant neoplasm of other and unspecified parts of biliary tract	1 (1.0%)
C25	Malignant neoplasm of pancreas	3 (2.9%)
C32	Malignant neoplasm of larynx	19 (18.3%)
C34	Malignant neoplasm of bronchus and lung	4 (3.8%)
C50	Malignant neoplasm of breast	9 (8.7%)
C54	Malignant neoplasm of corpus uteri	2 (1.9%)
C56	Malignant neoplasm of ovary	9 (8.7%)
C61	Malignant neoplasm of prostate	8 (7.7%)

**Table 4:** Nutritional characteristics of the studied group of cancer patients.

Characteristics	Number (%)
Weight loss	
Yes	69 (66.3%)
No	35 (33.7%)
Reduced food intake	
Yes	59 (56.7%)
No	45 (43.3%)
Nutritional support	
Enteral nutrition	25 (24%)
No enteral nutrition	79 (76%)
Global Health Status (QoL) ^1^	
High quality of life (score > 50)	31 (29.8%)
Low quality of life (score ≤ 50)	73 (70.2%)
Subjective Global Assessment (SGA)	
A (well-nourished)	28 (26.9%)
B (moderately malnourished or suspected of being malnourished)	48 (46.2%)
C (severely malnourished)	28 (26.9%)

^1^ Optimal cut points for global health status in EORTC QLQ-C30 [34].

**Table 5:** Sociodemographic and health-related characteristics of the studied group of cancer patients stratified by Subjective Global Assessment category.

Characteristics		Subjective Global Assessment Category			Cramer’s V	p *
		A (Well-Nourished)	B (Moderately Malnourished or Suspected of Being Malnourished)	C (Severely Malnourished)		
Sex	Woman *n* = 55	12 (42.9%)	27 (56.3%)	16 (57.1%)	0.122	0.4603
	Man *n* = 49	16 (57.1%)	21 (43.8%)	12 (42.9%)		
Education background	Higher *n* = 47	16 (57.1%)	22 (45.8%)	9 (32.1%)	0.210	0.2248
	General secondary *n* = 36	8 (28.6%)	14 (29.2%)	14 (50%)		
	Vocational secondary and primary *n* = 21	4 (14.3%)	12 (25%)	5 (17.9%)		
Employment	Full-time permanent *n* = 27	10 (35.7%)	11 (22.9%)	6 (21.4%)	0.174	0.7909
	Part-time permanent *n* = 4	1 (3.6%)	2 (4.2%)	1 (3.6%)		
	Full or parti-time temporary *n* = 5	0 (0%)	4 (8.3%)	1 (3.6%)		
	Pension *n* = 7	2 (7.1%)	4 (8.3%)	1 (3.6%)		
	Retirement *n* = 49	13 (46.4%)	22 (45.8%)	14 (50%)		
	Not employed *n* = 12	2 (7.1%)	5 (10.4%)	5 (17.9%)		
Place of residence	City over 500,000 inhabitants *n* = 31	12 (42.9%)	14 (29.2%)	5 (17.9%)	0.289	0.0262
	City 100,000 to 500,000 inhabitants *n* = 5	2 (7.1%)	2 (4.2%)	1 (3.6%)		
	City 20,000 to 100,000 inhabitants *n* = 21	4 (14.3%)	11 (22.9%)	6 (21.4%)		
	Town up to 20,000 inhabitants *n* = 13	7 (25%)	5 (10.4%)	1 (3.6%)		
	Rural area *n* = 34	3 (10.7%)	16 (33.3%)	15 (53.6%)		
Economic status	Good *n* = 25	10 (35.7%)	9 (18.8%)	6 (21.4%)	0.317	0.0073
	Rather good *n* = 40	13 (46.4%)	23 (47.9%)	4 (14.3%)		
	Rather bad *n* = 17	2 (7.1%)	5 (10.4%)	10 (35.7%)		
	Bad *n* = 10	0 (0%)	6 (12.5%)	4 (14.3%)		
	Difficult to say *n* = 12	3 (10.7%)	5 (10.4%)	4 (14.3%)		
Weight loss	Weight loss *n* = 69	3 (10.7%)	40 (83.3%)	26 (92.9%)	0.719	<0.0001
	No reported weight loss *n* = 35	25 (89.3%)	8 (16.7%)	2 (7.1%)		
Reduction in food intake	Reduction in food intake *n* = 59	4 (14.3%)	32 (66.7%)	23 (82.1%)	0.536	<0.0001
	No reported reduction in food intake *n* = 45	24 (85.7%)	16 (33.3%)	5 (17.9%)		
BMI category	Underweight *n* = 11	1 (3.6%)	5 (17.9%)	5 (17.9%)	0.245	0.0140
	Normal body weight *n* = 50	8 (28.6%)	28 (58.3%)	14 (50%)		
	Overweight *n* = 43	19 (67.9%)	15 (31.3%)	9 (32.1%)		
Nutritional support	No enteral nutrition *n* = 79	27 (34.2%)	39 (49.5%)	13 (16.5%)	0.444	<0.0001
	Enteral nutrition *n* = 25	1 (4.0%)	9 (36.0%)	15 (60.0%)		
Global Health Status (QoL)	High quality of life (score > 50) *n* = 31	15 (53.6%)	14 (29.2)	2 (7.1%)	0.336	0.0007
	Low quality of life (score ≤ 50) *n* = 73	13 (46.4%)	34 (70.8%)	26 (92.9%)		

* A Chi-squared test was applied.

**Table 6:** The summary of the descriptive statistics and reliability metrics for scales and individual items of the EORTC QLQ-INFO25 questionnaire.

	Mean ± SD	Median (IQR)	% of Ceiling Effect ^2^	% of Floor Effect ^3^	Cronbach’s Alpha ^4^
Information scales/items					
Information about the disease	58.25 ± 22.95	58.33 (25) ^1^	1.0	10.6	0.85
Information about medical tests	64.32 ± 25.57	66.67 (44.45) ^1^	1.0	21.2	0.90
Information about treatments	50.86 ± 21.74	55.56 (33.34) ^1^	1.9	3.8	0.85
Information about other services	29.73 ± 25.65	25 (41.67) ^1^	17.3	2.9	0.81
Information about different places of care	29.81 ± 31.48	33.33 (66.67) ^1^	44.2	5.8	-
Information about things patient can do to help themselves	35.9 ± 32.41	33.33 (66.67) ^1^	34.6	8.7	-
Written information	44.23 ± 49.91	0 (100)^1^	-	-	-
Information on CD/video	16.35 ± 37.16	0 (0)^1^	-	-	-
Satisfaction scales/items					
Satisfaction with the amount of information received	44.87 ± 22.17	33.33 (33.34) ^1^	10.6	0.0	-
Wish to receive more information	32.69 ± 47.14	0 (100) ^1^	-	-	-
Wish to receive less information	94.23 ± 23.43	100 (0) ^1^	-	-	-
Overall the information has been helpful	53.53 ± 19.39	66.67 (33.34) ^1^	1.0	3.8	-
Global Score	46.23 ± 15.71	45.37 (20.72)	0.0	0.0	0.92

^1^ The non-parametric distribution (Shapiro–Wilk test; *p* < 0.05); ^2^ ceiling values are not applicable for questions with a dichotomous nature; ^3^ floor values are not applicable for questions with a dichotomous nature; ^4^ Cronbach’s alpha is not applicable for single-item scales; SD—standard deviation; IQR—interquartile range.

**Table 7:** Number and percentage of patients reporting low satisfaction on the EORTC QLQ-INFO25 scales.

Scale Name	Scale Description	Number of Patients Declared Low Satisfaction	Percentage (%)
INFODIS	Information about the disease	32	30.8
INFOMEDT	Information about medical tests	31	29.8
INFOTREAT	Information about treatments	39	37.5
INFOTHSE	Information about other services	77	74.0
INFODIFP	Information about different places of care	75	72.1
INFOHELP	Information about things you can do to help yourself	69	66.3

**Table 8:** The scales and individual items of the EORTC QLQ-INFO25 questionnaire of the studied group of cancer patients stratified by sex.

	Male (*n* = 49)		Female (*n* = 55)		Cohen’s d	*p* **
	Mean ± SD	Median (IQR)	Mean ± SD	Median (IQR)		
Information scales/items						
Information about the disease	63.61 ± 20.18	66.67 (16.67) *	53.49 ± 24.36	50 (33.34) *	0.45	0.0192
Information about medical tests	68.03 ± 24.49	66.67 (33.33) *	61.01 ± 26.27	66.67 (33.34) *	0.28	0.2146
Information about treatments	56.01 ± 19.11	55.56 (27.78)	46.26 ± 23.06	50 (33.33)	0.46	0.0217
Information about other services	35.03 ± 28.05	33.33 (33.33) *	25.0 ± 22.51	16.67 (33.34) *	0.39	0.0691
Information about different places of care	38.78 ± 33.57	33.33 (66.67) *	21.82 ± 27.38	0 (33.33) *	0.55	0.0079
Information about things patient can do to help themselves	40.82 ± 30.63	33.33 (66.67) *	31.51 ± 33.59	33.33 (66.67) *	0.29	0.0905
Written information	38.78 ± 49.23	0 (100) *	49.09 ± 50.45	0 (100) *	0.21	0.2927
Information on CD/video	16.33 ± 37.34	0 (0) *	16.36 ± 37.34	0 (0) *	0.00	0.9959
Satisfaction scales/items						
Satisfaction with the amount of information received	51.02 ± 19.37	66.67 (33.34) *	39.39 ± 23.21	33.33 (33.34) *	0.54	0.0090
Wish to receive more information	42.86 ± 50.0	0 (100) *	23.64 ± 42.88	0 (0) *	0.41	0.0379
Wish to receive less information	91.84 ± 27.66	100 (0) *	96.36 ± 18.89	100 (0) *	0.19	0.3253
Overall the information has been helpful	55.78 ± 15.8	66.67 (33.34) *	51.51 ± 22.06	33.33 (33.34) *	0.22	0.1638
Global Score	49.91 ± 14.0	46.76 (15.97)	42.95 ± 16.54	40.97 (23.38)	0.45	0.0236

* The non-parametric distribution (Shapiro–Wilk test; *p* < 0.05); ** test *t*-Student applied (for normal distribution) or Mann–Whitney U test applied (for non-normal distribution); SD—standard deviation; IQR—interquartile range.

**Table 9:** The scales and individual items of the EORTC QLQ-INFO25 questionnaire of the studied group of cancer patients stratified by enteral nutrition applied.

	Enteral Nutrition (*n* = 25)		No Enteral Nutrition (*n* = 79)		Cohen’s d	*p* **
	Mean ± SD	Median (IQR)	Mean ± SD	Median (IQR)		
Information scales/items						
Information about the disease	63.67 ± 22.68	66.67 (16.67) *	56.54 ± 22.91	58.33 (25) *	0.31	0.0994
Information about medical tests	71.56 ± 19.27	66.67 (11.11) *	62.03 ± 26.96	66.67 (44.45) *	0.41	0.0833
Information about treatments	55.11 ± 21.27	55.56 (27.78)	49.51 ± 21.85	55.56 (33.34) *	0.26	0.2769
Information about other services	33.0 ± 25.17	33.33 (25)	28.69 ± 25.87	25.0 (41.67)	0.17	0.4669
Information about different places of care	34.67 ± 28.02	33.33 (66.67) *	28.27 ± 32.51	33.33 (66.67) *	0.21	0.2351
Information about things patient can do to help themselves	38.67 ± 29.94	33.33 (33.34) *	35.02 ± 33.29	33.33 (66.67) *	0.08	0.5419
Written information	48 ± 50.99	0 (100) *	43.04 ± 49.83	0 (100) *	0.10	0.6648
Information on CD/video	24 ± 43.59	0 (0) *	13.92 ± 34.84	0 (0) *	0.26	0.2373
Satisfaction scales/items						
Satisfaction with the amount of information received	53.33 ± 16.67	66.67 (33.34) *	42.19 ± 23.09	33.33 (33.34) *	0.55	0.0376
Wish to receive more information	52.0 ± 50.99	100 (100) *	26.58 ± 44.46	0 (100) *	0.53	0.0188
Wish to receive less information	100 ± 0.0	100 (0) *	92.41 ± 26.66	100 (0) *	0.40	0.1578
Overall the information has been helpful	57.33 ± 18.06	66.67 (33.34) *	52.32 ± 19.75	66.67 (33.34) *	0.26	0.2464
Global Score	52.61 ± 13.08	52.78 (15.28)	44.21 ± 16.0	41.67 (20.14) *	0.57	0.0045

* The non-parametric distribution (Shapiro–Wilk test; *p* < 0.05); ** test *t*-Student applied (for normal distribution) or Mann–Whitney U test applied (for non-normal distribution); SD—standard deviation; IQR—interquartile range.

**Table 10:** The scales and individual items of the EORTC QLQ-INFO25 questionnaire of the studied group of cancer patients stratified by age.

	19–49 Years (*n* = 27)		50–69 Years (*n* = 50)		70–82 Years (*n* = 27)		η^2^	*p* **
	Mean ± SD	Median (IQR)	Mean ± SD	Median (IQR)	Mean ± SD	Median (IQR)		
Information scales/items								
Information about								
… the disease	54.01 ± 24.72	50 (33.34) *	55.83 ± 22.16	58.33 (25) *	66.98 ± 20.99	66.67 (25) *	0.052	0.0537
… medical tests	57.61 ± 28.59	66.67 (44.45) *^,a^	61.78 ± 24.4	66.67 (22.23) *^,a^	75.72 ± 21.36	66.67 (33.33) *^,b^	0.075	0.0255
… treatments	48.77 ± 26.26	50 (44.45)	49.89 ± 20.62	55.56 (27.78)	54.73 ± 18.98	55.56 (33.34)	0.012	0.8365
… other services	23.15 ± 23.94	16.67 (25) *	30.33 ± 27.03	25 (41.67) *	35.19 ± 24.06	33.33 (41.66)	0.029	0.1297
… different places of care	23.46 ± 31.78	0 (33.33) *	30.67 ± 30.0	33.33 (66.67) *	34.57 ± 33.95	33.33 (66.67) *	0.017	0.3618
… things patient can do to help themselves	32.1 ± 32.66	33.33 (33.33) *	32.67 ± 28.96	33.33 (66.67) *	45.68 ± 37.15	33.33 (66.67) *	0.032	0.2615
Written information	44.44 ± 50.64	0 (100) *	48 ± 50.47	0 (100) *	37.04 ± 49.21	0 (100) *	0.008	0.6549
Information on CD/video	11.11 ± 32.03	0 (0) *	22 ± 41.85	0 (0) *	11.11 ± 32.03	0 (0) *	0.022	0.3280
Satisfaction scales/items								
Satisfaction with the amount of information received	35.8 ± 22.51	33.33 (33.34) *^a^	45.33 ± 22.09	33.33 (33.34) *^,a^	53.09 ± 19.08	66.67 (33.34) *^,b^	0.080	0.0153
Wish to receive more information	29.63 ± 46.53	0 (100) *	32 ± 47.12	0 (100) *	37.04 ± 49.21	0 (100) *	0.003	0.8377
Wish to receive less information	92.59 ± 26.69	100 (0) *	96 ± 19.79	100 (0) *	92.59 ± 26.69	100 (0) *	0.005	0.7599
Overall the information has been helpful	46.91 ± 23.13	33.33 (33.34) *	56 ± 18.37	66.67 (33.34) *	55.56 ± 16.02	66.67 (33.34) *	0.041	0.0847
Global Score	41.63 ± 17.1	40.28 (16.44) *	46.71 ± 14.79	47.11 (21.3)	49.94 ± 15.38	48.38 (24.53)	0.038	0.0715

* The non-parametric distribution (Shapiro–Wilk test; *p* < 0.05); ** analysis of variance (ANOVA) test (for normal distribution) or Kruskall–Wallis ANOVA test (for non-normal distribution); different letters in rows (^a,b^) indicate significant differences between groups (*p* < 0.05)—Tukey’s post hoc analysis; SD—standard deviation; IQR—interquartile range.

**Table 11:** The scales and individual items of the EORTC QLQ-INFO25 questionnaire of the studied group of cancer patients stratified by Subjective Global Assessment (SGA).

	A (Well-Nourished) (*n* = 28)		B (Moderately Malnourished or Suspected of Being Malnourished) (*n* = 48)		C (Severely Malnourished) (*n* = 28)		η^2^	*p* **
	Mean ± SD	Median (IQR)	Mean ± SD	Median (IQR)	Mean ± SD	Median (IQR)		
Information scales/items								
Information about								
… the disease	60.71 ± 25.14	66.67 (37.5)	54.34 ± 21.05	50 (25) *	62.5 ± 23.52	62.5 (20.84)	0.026	0.2571
… medical tests	68.25 ± 26.66	66.67 (55.56) *	60.42 ± 25.72	66.67 (22.23) *	67.06 ± 24.1	66.67 (27.78)	0.020	0.4177
… treatments	53.18 ± 22.65	55.56 (30.56)	45.49 ± 19.44	50 (27.78) *	57.74 ± 22.95	55.56 (27.78)	0.059	0.0997
… other services	28.57 ± 24.89	25 (41.67) *	28.3 ± 24.96	16.67 (33.34) *	33.33 ± 28.05	29.17 (33.33)	0.007	0.7374
… different places of care	25 ± 34.7	0 (66.67) *	29.17 ± 30.46	33.33 (66.67) *	35.71 ± 29.99	33.33 (66.67)	0.016	0.2801
… things patient can do to help themselves	35.71 ± 36.21	33.33 (66.67) *	32.64 ± 28.76	33.33 (66.67) *	41.67 ± 34.7	33.33 (66.67)	0.013	0.5887
Written information	42.86 ± 50.4	0 (100) *	45.83 ± 50.35	0 (100) *	42.86 ± 50.4	0 (100)	0.000	0.9551
Information on CD/video	10.71 ± 31.5	0 (0) *	18.75 ± 39.44	0 (0) *	17.86 ± 39	0 (0)	0.009	0.6407
Satisfaction scales/items								
Satisfaction with the amount of information received	45.24 ± 22.62	33.33 (33.34) *	40.28 ± 23.78	33.33 (33.34) *	52.38 ± 16.8	66.67 (33.34)	0.051	0.1011
Wish to receive more information	35.71 ± 48.8	0 (100) *	29.17 ± 45.93	0 (100) *	35.71 ± 48.8	0 (100)	0.005	0.7793
Wish to receive less information	89.29 ± 31.5	100 (0) *	95.83 ± 20.19	100 (0) *	96.43 ± 18.9	100 (0)	0.017	0.4235
Overall the information has been helpful	51.19 ± 21.24	33.33 (33.34) *	53.47 ± 19.13	66.67 (33.34) *	55.95 ± 18.27	66.67 (33.34)	0.008	0.5374
Global Score	45.54 ± 16.91	41.9 (13.31) *	44.47 ± 15.2	45.61 (24.78)	49.93 ± 15.27	51.04 (20.61)	0.021	0.3377

* The non-parametric distribution (Shapiro–Wilk test; *p* < 0.05); ** analysis of variance (ANOVA) test (for normal distribution) or Kruskall–Wallis ANOVA test (for non-normal distribution); SD—standard deviation; IQR—interquartile range.

## Data Availability

The data presented in this study are available on request from the corresponding author. The data are not publicly available due to ethical restrictions.

## Supplementary Materials

The following supporting information can be downloaded at: https://www.mdpi.com/article/10.3390/jcm14030697/s1, Table S1: The correlations between scales and individual items of the EORTC QLQ-INFO25 questionnaire in the studied group of cancer patients; Table S2: The correlations between scales and individual items of the EORTC QLQ-INFO25 and the EORTC QLQ-C30 questionnaire in the studied group of cancer patients.

## References

[1] Ferlay J., Ervik M., Lam F., Laversanne M., Colombet M., Mery L., Piñeros M., Znaor A., Soerjomataram I., Bray F. (2024). Global Cancer Observatory: Cancer Today.

[2] Argiles J.M. (2005). Cancer-associated malnutrition. Eur. J. Oncol. Nurs..

[3] Arends J. (2024). Malnutrition in cancer patients: Causes, consequences and treatment options. Eur. J. Surg. Oncol..

[4] Pradeep R., Deora K., Danca E., Young T., Mott K., Chezik A., Kudelka A.P. (2024). Unveiling the impact: Exploring malnutrition rates in patients with cancer and its association with anticancer treatments. J. Clin. Oncol..

[5] Bossi P., Delrio P., Mascheroni A., Zanetti M. (2021). The spectrum of malnutrition/cachexia/sarcopenia in oncology according to different cancer types and settings: A narrative review. Nutrients.

[6] Muscaritoli M., Arends J., Bachmann P., Baracos V., Barthelemy N., Bertz H., Bischoff S.C. (2021). ESPEN practical guideline: Clinical Nutrition in cancer. Clin. Nutr..

[7] Aprile G., Basile D., Giaretta R., Schiavo G., La Verde N., Corradi E., Stragliotto S. (2021). The clinical value of nutritional care before and during active cancer treatment. Nutrients.

[8] Shrestha A., Martin C., Burton M., Walters S., Collins K., Wyld L. (2019). Quality of life versus length of life considerations in cancer patients: A systematic literature review. Psychooncology.

[9] Ramasubbu S.K., Pasricha R.K., Nath U.K., Rawat V.S., Das B. (2021). Quality of life and factors affecting it in adult cancer patients undergoing cancer chemotherapy in a tertiary care hospital. Cancer Rep..

[10] Efficace F., Collins G.S., Cottone F., Giesinger J.M., Sommer K., Anota A., Schlussel M.M., Fazi P., Vignetti M. (2021). Patient-Reported Outcomes as Independent Prognostic Factors for Survival in Oncology: Systematic Review and Meta-Analysis. Value Health.

[11] Carr A.J., Gibson B., Robinson P.G. (2001). Measuring quality of life: Is quality of life determined by expectations or experience?. BMJ.

[12] Abelsson T., Morténius H., Bergman S., Karlsson A.K. (2020). Quality and availability of information in primary healthcare: The patient perspective. Scand. J. Prim. Health Care.

[13] Arraras J.I., Greimel E., Sezer O., Chie W.C., Bergenmar M., Costantini A., Young T., Vlasic K.K., Velikova G. (2010). An international validation study of the EORTC QLQ-INFO25 questionnaire: An instrument to assess the information given to cancer patients. Eur. J. Cancer.

[14] Bezerra M., Domenico E.B.L.D. (2024). Cancer patient satisfaction regarding the quality of information received: Psychometric validity of EORTC QLQ-INFO25. Rev. Bras. Enferm..

[15] Tabchi S., El Rassy E., Khazaka A., El Karak F., Kourie H.R., Chebib R., Assi T., Ghor M., Naamani L., Richa S. (2016). Validation of the EORTC QLQ-INFO25 questionnaire in Lebanese cancer patients: Is ignorance a Bliss?. Qual. Life Res..

[16] Nationwide Cancer Database. https://onkologia.org.pl/en.

[17] EORTC Quality of Life. https://qol.eortc.org/questionnaire/eortc-qlq-c30/.

[18] Cocks K., Wells J.R., Johnson C., Schmidt H., Koller M., Oerlemans S., Velikova G., Pinto M., Tomaszewski K.A., Aaronson N.K. (2023). Content validity of the EORTC quality of life questionnaire QLQ-C30 for use in cancer. Eur. J Cancer..

[19] Tomaszewski K.A., Püsküllüoğlu M., Biesiada K., Bochenek J., Nieckula J., Krzemieniecki K. (2013). Validation of the polish version of the eortc QLQ-C30 and the QLQ-OG25 for the assessment of health-related quality of life in patients with esophagi-gastric cancer. J. Psychosoc. Oncol..

[20] Szadowska-Szlachetka Z., Stanisławek A., Charzyńska-Gula M., Kachaniuk H., Muzyczka K., Kocka K. (2013). Differences in the quality of life of women before and after breast reconstruction measured with the use of EORTC QLQ-C 30 and EORTC QLQ-BR 23 scale. Menopause Rev..

[21] Głowacka I., Zegarski W., Hagner W., Nowacka K., Nowikiewicz T. (2015). Quality of Life Evaluation in Women with Breast Cancer Undergoing BCT with Sentinel Lymph Node Biopsy. Polskie Forum Psychologiczne.

[22] Quality of Life. https://qol.eortc.org/questionnaires.

[23] Giesinger J.M., Kieffer J.M., Fayers P.M., Groenvold M., Petersen M.A., Scott N.W., Sprangers M.A., Velikova G., Aaronson N.K., EORTC Quality of Life Group (2016). Replication and validation of higher order models demonstrated that a summary score for the EORTC QLQ-C30 is robust. J. Clin. Epidemiol..

[24] Groenvold M., Klee M.C., Sprangers M.A., Aaronson N.K. (1997). Validation of the EORTC QLQ-C30 quality of life questionnaire through combined qualitative and quantitative assessment of patient-observer agreement. J. Clin. Epidemiol..

[25] (2022). Selpercatinib (Retevmo): CADTH Reimbursement Review: Therapeutic Area: Thyroid Cancer [Internet].

[26] EORTC QLQ-C30 Scoring Manual. https://www.eortc.org/app/uploads/sites/2/2018/02/SCmanual.pdf.

[27] EORTC QLQ-INFO25 Questionnaire. https://qol.eortc.org/questionnaire/qlq-info25.

[28] Püsküllüoğlu M., Tomaszewski K.A., Zygulska A.L., Ochenduszko S., Streb J., Tomaszewska I.M., Krzemieniecki K. (2014). Pilot Testing and Preliminary Psychometric Validation of the Polish Translation of the EORTC INFO25 Questionnaire: Validation of the Polish version of INFO25-pilot study. Appl. Res. Qual. Life.

[29] EORTC QLQ-INFO25. https://www.eortc.org/app/uploads/sites/2/2018/08/Specimen-INFO25-English-1.1.pdf.

[30] Pinto A.C., Ferreira-Santos F., Lago L.D., de Azambuja E., Pimentel F.L., Piccart-Gebhart M., Razavi D. (2014). Information perception, wishes, and satisfaction in ambulatory cancer patients under active treatment: Patient-reported outcomes with QLQ-INFO25. Ecancermedicalscience.

[31] Fayers P.M. (2001). EORTC QLQ-INFO25 Scoring Manual.

[32] A Healthy Lifestyle—WHO Recommendations. https://www.who.int/europe/news-room/fact-sheets/item/a-healthy-lifestyle---who-recommendations.

[33] da Silva Fink J., Daniel de Mello P., Daniel de Mello E. (2015). Subjective global assessment of nutritional status—A systematic review of the literature. Clin. Nutr..

[34] Detsky A.S., McLaughlin J.R., Baker J.P., Johnston N., Whittaker S., Mendelson R.A., Jeejeebhoy K.N. (1987). What is subjective global assessment of nutritional status?. JPEN J. Parenter. Enter. Nutr..

[35] Diouf M., Bonnetain F., Barbare J.C., Bouché O., Dahan L., Paoletti X., Filleron T. (2015). Optimal cut points for quality of life questionnaire-core 30 (QLQ-C30) scales: Utility for clinical trials and updates of prognostic systems in advanced hepatocellular carcinoma. Oncologist.

[36] Ugai T., Sasamoto N., Lee H.Y., Ando M., Song M., Tamimi R.M., Kawachi I., Campbell P.T., Giovannucci E.L., Weiderpass E. (2022). Is early-onset cancer an emerging global epidemic? Current evidence and future implications. Nat. Rev. Clin. Oncol..

[37] Van Herck Y., Feyaerts A., Alibhai S., Papamichael D., Decoster L., Lambrechts Y., Pinchuk M., Bechter O., Herrera-Caceres J., Bibeau F. (2021). Is cancer biology different in older patients?. Lancet Healthy Longev..

[38] Arslan J., Benke K. (2023). Statistical Analysis of Ceiling and Floor Effects in Medical Trials. Appl. Biosci..

[39] Morera O.F., Stokes S.M. (2016). Coefficient α as a Measure of Test Score Reliability: Review of 3 Popular Misconceptions. Am. J. Public Health..

[40] Gulledge C.M., Smith D.G., Ziedas A., Muh S.J., Moutzouros V., Makhni E.C. (2019). Floor and Ceiling Effects, Time to Completion, and Question Burden of PROMIS CAT Domains Among Shoulder and Knee Patients Undergoing Nonoperative and Operative Treatment. JBJS Open Access.

[41] DeVellis R.F. (1991). Scale Development.

[42] Rovetta A. (2020). Raiders of the Lost Correlation: A Guide on Using Pearson and Spearman Coefficients to Detect Hidden Correlations in Medical Sciences. Cureus.

[43] Cohen J. (1988). Statistical Power Analysis for the Behavioral Sciences.

[44] Sapra R.L., Saluja S. (2021). Understanding statistical association and correlation. Curr. Med. Res. Pr..

[45] de van der Schueren M.A.E., Laviano A., Blanchard H., Jourdan M., Arends J., Baracos V.E. (2018). Systematic review and meta-analysis of the evidence for oral nutritional intervention on nutritional and clinical outcomes during chemo(radio)therapy: Current evidence and guidance for design of future trials. Ann. Oncol..

[46] Muscaritoli M., Lucia S., Farcomeni A., Lorusso V., Saracino V., Barone C., Plastino F., Gori S., Magarotto R., Carteni G. (2017). Prevalence of malnutrition in patients at first medical oncology visit: The PreMiO study. Oncotarget.

[47] Hager K.K. (2016). Management of Weight Loss in People With Cancer. J. Adv. Pract. Oncol..

[48] Rios T.C., de Oliveira L.P.M., da Costa M.L.V., da Silva Baqueiro Boulhosa R.S., Roriz A.K.C., Ramos L.B., Bueno A.A. (2021). A poorer nutritional status impacts quality of life in a sample population of elderly cancer patients. Health Qual. Life Outcomes.

[49] Sánchez-Torralvo F.J., Contreras-Bolívar V., Ruiz-Vico M., Abuín-Fernández J., González-Almendros I., Barrios M., Olveira G. (2022). Relationship between malnutrition and the presence of symptoms of anxiety and depression in hospitalized cancer patients. Support. Care Cancer.

[50] Ni J., Zhang L. (2020). Cancer Cachexia: Definition, Staging, and Emerging Treatments. Cancer Manag. Res..

[51] Fearon K., Strasser F., Anker S.D., Bosaeus I., Bruera E., Fainsinger R.L., Jatoi A., Loprinzi C., MacDonald N., Mantovani G. (2011). Definition and classification of cancer cachexia: An international consensus. Lancet Oncol..

[52] Bozzetti F., Mariani L. (2009). Defining and classifying cancer cachexia: A proposal by the SCRINIO Working Group. JPEN J. Parenter. Enter. Nutr..

[53] Evans W.J., Morle J.E., Argilés J., Bales C., Baracos V., Guttridge D., Jatoi A., Kalantar-Zadeh K., Lochs H., Mantovani G. (2008). Cachexia: A new definition. Clin. Nutr..

[54] Cruz-Jentoft A.J., Bahat G., Bauer J., Boirie Y., Bruyère O., Cederholm T., Cooper C., Landi F., Rolland Y., Sayer A.A. (2019). Sarcopenia: Revised European consensus on definition and diagnosis. Age Ageing.

[55] Prado C.M., Gonzalez M.C., Heymsfield S.B. (2015). Body composition phenotypes and obesity paradox. Curr. Opin. Clin. Nutr. Metab. Care.

[56] Wen H., Deng G., Shi X., Liu Z., Lin A., Cheng Q., Zhang J., Luo P. (2024). Body mass index, weight change, and cancer prognosis: A meta-analysis and systematic review of 73 cohort studies. ESMO Open.

[57] Landi F., Camprubi-Robles M., Bear D., Cederholm T., Malafarina V., Welch A., Cruz-Jentoft A. (2019). Muscle loss: The new malnutrition challenge in clinical practice. Clin. Nutr..

[58] Evans W.J. (2010). Skeletal muscle loss: Cachexia, sarcopenia, and inactivity. Am. J. Clin. Nutr..

[59] Cederholm T., Jensen G.L., Correia M.I.T.D., Gonzalez M.C., Fukushima R., Higashiguchi T., Baptista G., Barazzoni R., Blaauw R., Coats A. (2019). GLIM criteria for the diagnosis of malnutrition—A consensus report from the global clinical nutrition community. Clin. Nutr..

[60] Kiss N., Prado C.M., Daly R.M., Denehy L., Edbrooke L., Baguley B.J., Fraser S.F., Khosravi A., Abbott G. (2023). Low muscle mass, malnutrition, sarcopenia, and associations with survival in adults with cancer in the UK Biobank cohort. J. Cachexia Sarcopenia Muscle.

[61] Baldwin C., Spiro A., Ahern R., Emery P.W. (2012). Oral nutritional interventions in malnourished patients with cancer: A systematic review and meta-analysis. J. Natl. Cancer. Inst..

[62] Bourdel-Marchasson I., Blanc-Bisson C., Doussau A., Germain C., Blanc J.F., Dauba J., Lahmar C., Terrebonne E., Lecaille C., Ceccaldi J. (2014). Nutritional advice in older patients at risk of malnutrition during treatment for chemotherapy: A two-year randomized controlled trial. PLoS ONE.

[63] Parsons H.M., Forte M.L., Abdi H.I., Brandt S., Claussen A.M., Wilt T., Klein M., Ester E., Landsteiner A., Shaukut A. (2023). Nutrition as prevention for improved cancer health outcomes: A systematic literature review. JNCI Cancer Spectr..

[64] Cotogni P., Stragliotto S., Ossola M., Collo A., Riso S., Intersociety Italian Working Group for Nutritional Support in Cancer (2021). The role of nutritional support for cancer patients in palliative care. Nutrients.

[65] Ashok A., Niyogi D., Ranganathan P., Tandon S., Bhaskar M., Karimundackal G., Pramesh C.S. (2020). The enhanced recovery after surgery (ERAS) protocol to promote recovery following esophageal cancer resection. Surg. Today.

[66] Lee H.Y., Jin S.W., Henning-Smith C., Lee J., Lee J. (2021). Role of Health Literacy in Health-Related Information-Seeking Behavior Online: Cross-sectional Study. J. Med. Internet Res..

[67] Heizomi H., Iraji Z., Vaezi R., Bhalla D., Morisky D.E., Nadrian H. (2020). Gender Differences in the Associations Between Health Literacy and Medication Adherence in Hypertension: A Population-Based Survey in Heris County, Iran. Vasc. Health Risk Manag..

[68] Wang M.P., Viswanath K., Lam T.H., Wang X., Chan S.S. (2013). Social determinants of health information seeking among Chinese adults in Hong Kong. PLoS ONE.

[69] Bergenmar M., Johansson H., Sharp L. (2014). Patients’ perception of information after completion of adjuvant radiotherapy for breast cancer. Eur. J. Oncol. Nurs..

[70] McCaughan E., McKenna H. (2007). Information-seeking behaviour of men newly diagnosed with cancer: A qualitative study. J. Clin. Nurs..

[71] Arraras J.I., Manterola A., Hernández B., Arias de la Vega F., Martínez M., Vila M., Eito C., Vera R., Domínguez M.Á. (2011). The EORTC information questionnaire, EORTC QLQ-INFO25. Validation study for Spanish patients. Clin. Transl. Oncol..

