# Supplementary material for: Nutritional Status and Information Provided to Polish Cancer Patients Assessed Using the EORTC QLQ-INFO25 Questionnaire
Source: J Clin Med. 2025 Jan 22;14(3):697. doi: 10.3390/jcm14030697 (PMC11818191; doi:10.3390/jcm14030697)
Supplement: Supplementary file 1 [file jcm-14-00697-s001.zip › jcm-3379762-supplementary.pdf]

Journal of Clinical Medicine

MDPI

Supplementary

Nutritional Status and Information Provided to Polish Cancer Patients Assessed using the EORTC QLQ-INFO25 Questionnaire

Elwira Gliwska [1,2*], Dominika Głąbska [3] Zuzanna Zaczek [4,5], Jacek Sobocki [5†] and Dominika Guzek [1†]

[1] Department of Food Market and Consumer Research, Institute of Human Nutrition Sciences, Warsaw University of Life Sciences (WULS-SGGW), 159C Nowoursynowska Street, 02-776 Warsaw, Poland
[2] Cancer Epidemiology and Primary Prevention Department, Maria Sklodowska-Curie National Research Institute of Oncology, 15B Wawelska Street, 02-034 Warsaw, Poland
[3] Department of Dietetics, Institute of Human Nutrition Sciences, Warsaw University of Life Sciences (WULS-SGGW), 159C Nowoursynowska Street, 02-776 Warsaw, Poland
[4] Department of Human Nutrition, Faculty of Health Sciences, Medical University of Warsaw, 27 Erazma Ciolka Street, 01-445 Warsaw, Poland
[5] Department of General Surgery and Clinical Nutrition, Centre of Postgraduate Medical Education in Warsaw, 231 Czerniakowska Street, 00-416 Warsaw, Poland
* Author to whom correspondence should be addressed.
† These authors contributed equally to this work.

Supplementary Table S1. The correlations between scales and individual items of the EORTC QLQ-INFO25 questionnaire in the studied group of cancer patients.

	INFODIS	INFOMEDT	INFOTREAT	INFOTHSE	INFODIFP	INFOHELP	INFOWRIN	INFOCD	SATINFO	RECMORE	RECLESS	OVERHELP	GLOBAL SCORE
INFODIS	-												
INFOMEDT	0.67*	-											
INFOTREAT	0.79*	0.67*	-										
INFOTHSE	0.57*	0.37*	0.49*	-									
INFODIFP	0.36*	0.24*	0.33*	0.60*	-								
INFOHELP	0.48*	0.31*	0.45*	0.60*	0.67*	-							
INFOWRIN	0.09	0.10	0.11	0.10	0.01	0.09	-						
INFOCD	0.22*	0.08	0.10	0.06	0.00	0.00	0.13	-					
SATINFO	0.69*	0.59*	0.61*	0.58*	0.40*	0.42*	0.16	0.15	-				
RECMORE	0.25*	0.08	0.24*	0.24*	0.24*	0.31*	-0.13	0.02	0.18	-			
RECLESS	-0.17	-0.12	-0.15	-0.16	-0.04	-0.20*	0.14	-0.23*	-0.12	0.08	-		
OVERHELP	0.38*	0.47*	0.45*	0.34*	0.23*	0.22*	0.25*	0.17	0.59*	0.06	-0.03	-	
GLOBAL SCORE	0.75*	0.61*	0.72*	0.68*	0.60*	0.66*	0.39*	0.30*	0.76*	0.42*	-0.01	0.59*	-

* statistically significant correlation (p<0.05); INFODIS – Information about the disease; INFOMEDT – Information about medical tests; INFOTREAT – Information about treatments; INFOTHSE – Information about other services; INFODIFP – Information about different places of care; INFOHELP – Information about things patient can do to help themselves; INFOWRIN – Written information; INFOCD – Information on CD/video; SATINFO – Satisfaction with the information received; RECMORE – Wish to receive more information; RECLESS – Wish to receive less information; OVERHELP – Overall the information has been helpful; GLOBAL SCORE – The average of all the scales/single-item measures.

Supplementary Table S2. The correlations between scales and individual items of the EORTC QLQ-INFO25 and the EORTC QLQ-C30 questionnaire in the studied group of cancer patients.

	INFODIS	INFOMEDT	INFOTREAT	INFOTHSE	INFODIFP	INFOHELP	INFOWRIN	INFOCD	SATINFO	RECMORE	RECLESS	OVERHELP	GLOBAL SCORE
FS - Physical	-0.14	-0.14	-0.11	-0.10	-0.01	-0.06	0.08	0.05	-0.08	0.07	0.14	-0.17	-0.05
FS - Role	-0.17	-0.23*	-0.15	-0.15	-0.08	-0.10	-0.05	-0.01	-0.19	0.11	0.10	-0.22*	-0.19
FS - Cognitive	-0.09	-0.11	-0.12	-0.04	-0.01	-0.07	-0.01	-0.09	0.01	0.08	-0.02	-0.08	-0.08
FS - Emotional	0.01	-0.14	-0.08	0.13	0.21*	0.13	-0.05	-0.10	0.11	0.25*	-0.02	-0.02	0.06
FS - Social	-0.09	-0.23*	-0.12	0.05	0.14	0.10	-0.06	0.02	-0.02	0.15	0.04	-0.18	-0.03
SS - Fatigue	0.07	0.11	0.17	0.02	-0.01	-0.01	0.01	-0.05	-0.05	-0.10	-0.03	0.18	0.03
SS - Pain	0.18	0.21	0.16	0.14	0.03	0.01	0.03	0.01	0.18	-0.08	-0.06	0.29*	0.14
SS - Vomit-ing/nausea	0.18	0.17	0.24*	0.03	0.12	0.18	0.10	-0.03	0.10	-0.05	0.11	0.25*	0.20*
SQ - Dyspnoea	0.18	0.24*	0.22*	0.06	0.08	0.08	-0.07	-0.01	0.14	0.02	0.01	0.14	0.14
SQ - Insomnia	0.03	0.12	0.11	-0.06	-0.10	-0.13	-0.16	0.15	0.07	-0.02	-0.06	0.16	0.01
SQ - Appetite loss	0.13	0.20*	0.13	-0.02	0.00	0.03	-0.04	0.07	0.09	0.06	0.19	0.09	0.14
SQ - Constipation	0.06	-0.12	0.07	0.14	0.16	0.13	0.10	-0.02	0.07	0.11	0.04	0.11	0.14
SQ - Diarrhea	0.13	0.06	0.09	0.00	0.01	0.02	-0.05	-0.05	0.00	0.02	-0.12	-0.04	-0.04
SQ - Financial difficulties	0.03	0.00	0.08	0.08	-0.02	0.06	0.02	0.02	0.02	-0.04	0.02	0.11	0.05
Global Health Status/QoL	-0.07	-0.15	-0.02	-0.05	0.09	0.04	0.05	0.04	0.01	0.09	0.00	-0.07	0.00

* statistically significant correlation ($p<0.05$); INFODIS – Information about the disease; INFOMEDT – Information about medical tests; INFOTREAT – Information about treatments; INFOTHSE – Information about other services; INFODIFP – Information about different places of care; INFOHELP – Information about things patient can do to help themselves; INFOWRIN – Written information; INFOCD – Information on CD/video; SATINFO – Satisfaction with the information received; RECMORE – Wish to receive more information; RECLESS – Wish to receive less information; OVERHELP – Overall the information has been helpful; GLOBAL SCORE – The average of all the scales/single-item measures; FS – Functional Scales; SS – Symptom Scales; SQ – Single-item measures.
